# Chronic Allergen Challenge Induces Corticosteroid Insensitivity With Persistent Airway Remodeling and Type 2 Inflammation

**DOI:** 10.3389/fphar.2022.855247

**Published:** 2022-04-11

**Authors:** Brandon W. Lewis, Maria L. Ford, Aiman Q. Khan, Joshua Walum, Rodney D. Britt

**Affiliations:** ^1^ Center for Perinatal Research, The Abigail Wexner Research Institute at Nationwide Children’s Hospital, Columbus, OH, United States; ^2^ Department of Pediatrics, The Ohio State University, Columbus, OH, United States

**Keywords:** asthma, corticosteroids, airway remodeling, type 2 inflammation, airway hyperresponsiveness

## Abstract

Type 2-high severe asthma is described as a distinct endotype with Th2 inflammation, high eosinophil lung infiltration, impaired lung function, and reduced corticosteroid sensitivity. While the inflammatory milieu is similar to mild asthma, patients with type 2-high severe asthma likely have underlying mechanisms that sustain asthma pathophysiology despite corticosteroid treatments. Acute and chronic allergen models induce robust type 2 inflammatory responses, however differences in corticosteroid sensitivity remains poorly understood. In the present study, we sensitized and challenged mice with ovalbumin (OVA; acute model) or mixed allergens (MA; chronic model). Corticosteroid sensitivity was assessed by administering vehicle, 1, or 3 mg/kg fluticasone propionate (FP) and examining key asthmatic features such as airway inflammation, remodeling, hyperresponsiveness, and antioxidant capacity. Both acute and chronic allergen exposure exhibited enhanced AHR, immune cell infiltration, airway inflammation, and remodeling, but corticosteroids were unable to fully alleviate inflammation, AHR, and airway smooth muscle mass in MA-challenged mice. While there were no differences in antioxidant capacity, persistent IL-4+ Th2 cell population suggests the MA model induces type 2 inflammation that is insensitive to corticosteroids. Our data indicate that chronic allergen exposure is associated with more persistent type 2 immune responses and corticosteroid insensitivity. Understanding differences between acute and chronic allergen models could unlock underlying mechanisms related to type 2-high severe asthma.

## Introduction

Severe asthma is characterized by enhanced airway inflammation, remodeling, and airway hyperresponsiveness (AHR) that persist in the presence of corticosteroid treatment (Hansbro et al., 2017; Saglani, 2017). The airway inflammatory milieu or asthma endotype is thought to influence corticosteroid sensitivity (Ray et al., 2020). Type 2 inflammation is central to the pathogenesis of allergic asthma and consists of increased levels of Th2 cytokines (IL-4, IL-5, and IL-13), Th2 cell, ILC2, and eosinophil infiltration. Although type 2 inflammation is recognized as corticosteroid sensitive in patients with mild asthma (Berry et al., 2007; Woodruff et al., 2009), studies show that type 2 inflammation can be insensitive to corticosteroids in severe asthma (ten Brinke et al., 2004; Woodruff et al., 2009; Peters et al., 2019).

In type 2 inflammation, IL-4 and IL-13 secreted by Th2 and/or ILC2 cells promote airway inflammation. IL-4 and IL-13 induce AHR and airway remodeling, key characteristics that contribute to airway narrowing and airflow obstruction in human asthma (Kubo, 2017). Airway remodeling is greater in severe asthma, suggesting a role in corticosteroid insensitivity (Benayoun et al., 2003; Bourdin et al., 2007; Bossley et al., 2009). Individuals with type 2-high severe asthma demonstrate decreases in corticosteroid sensitivity, with studies implicating disruption in glucocorticoid receptor (GR) activity and persistently activated ILC2 populations (Chang et al., 2015; Liu et al., 2018). Type 2 inflammation has also been associated with increases in oxidative stress and decreased antioxidant capacity in asthma (Wang, 2013; Chan et al., 2016). Despite these findings, the underlying mechanisms of corticosteroid insensitivity in type 2-high severe asthma remains poorly understood.

Allergen sensitization and challenge in mice is a longstanding approach to understand the complex airway inflammation and pathophysiology associated with allergic asthma. Acute allergen models, such as the ovalbumin (OVA) model, induces robust Th2 airway inflammation and airway hyperresponsiveness. But its appropriateness for modeling human asthma is questionable as airway remodeling is not persistent (Kumar et al., 2008). In recent years, chronic allergen models have been commonly utilized to study type 2-mediated allergic airway inflammation. Here, mice are sensitized and challenged, via inhalation or aerosolization, for up to several weeks leading to robust type 2 inflammation and airway thickening or remodeling (Nials and Uddin, 2008).

Previous studies have investigated mechanisms related to airway inflammation, AHR, and remodeling in mouse models of allergic airway inflammation (Doras et al., 2018), but not in the context of corticosteroid sensitivity. In this study, we compared corticosteroid sensitivity in acute and chronic mouse models of allergic airway inflammation. We hypothesized that chronic allergen challenge would exhibit increased corticosteroid insensitivity with greater airway inflammation and remodeling. Although both acute and chronic models induced a similar immune cell infiltration and AHR, we found evidence of differences in corticosteroid sensitivity of type 2 inflammation. Our data show chronic allergen models induce corticosteroid insensitivity and comparisons to acute models could help identify mechanisms related to corticosteroid insensitivity in type 2 inflammation.

## Materials and Methods

**FIGURE 1:**
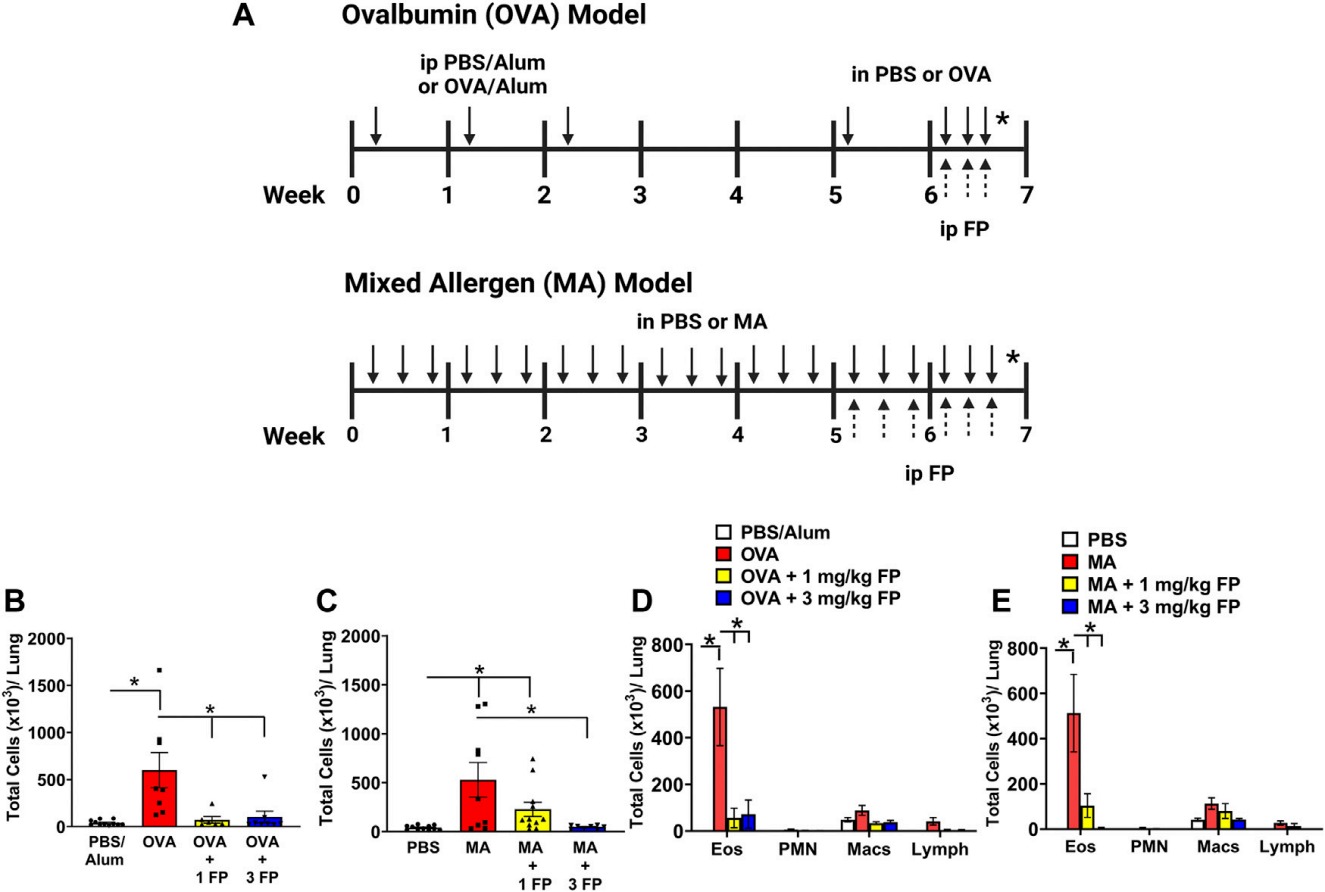
Corticosteroids reduce total airway immune cell and eosinophilic immune cell infiltration in acute and chronic allergic airway inflammation. **(A)** For acute allergen model, mice were sensitized with PBS or ovalbumin (OVA) and aluminum hydroxide adjuvant (alum), and then intranasally challenged with OVA. Mice were treated with either vehicle, 1 or 3 mg/kg fluticasone propionate (FP) by IP. For chronic allergen model, newborn mice were intranasally challenged with mixed allergen (MA) three times per week for 7 weeks. Mice were treated with either vehicle, 1 or 3 mg/kg FP by intraperitoneal injections 3 days a week during the last 2 weeks. Figure 1A was created using Biorender on 01/12/2022. **(B)** OVA- and **(C)** MA-challenged mice exhibited significantly higher total immune cell numbers in bronchoalveolar lavage. Total eosinophil numbers were significantly increased in **(D)** OVA- and **(E)** MA-challenged mice. Treatment with 1 mg/kg and 3 mg/kg FP significantly reduced total eosinophils in OVA- and MA-challenged mice. Data are presented as ± SEM, *n* = 6–11 mice per group. *p* < 0.05. * indicates significant difference between groups.

***Mouse Allergen Sensitization and Challenge.*** All mouse studies were approved by the Institutional Animal Care and Use Committee at Nationwide Children’s Hospital. C57BL/6J (stock no. 000664) mice were procured from Jackson Laboratory (Bar Harbor, ME). Mice were maintained on 12 h light-dark cycle within Research Building III at the Abigail Wexner Research Institute and provided food and water *ad libitum*. Adult male and female mice were bred to generate pup litters that were randomly assigned a treatment group. For acute exposure, newborn male and female mice were sensitized by intraperitoneal injections of either Dulbecco’s Phosphate Buffered Saline (DBPS) or 100 μg ovalbumin (OVA, cat no. vac-pova; InvivoGen, San Diego, CA) with aluminum hydroxide (Alum; cat no. 77161; Thermo Scientific, Rockford, IL) once a week during the first 3 weeks of life. During the sixth week, mice were challenged once intranasally with either DPBS or 100 μg OVA. During the seventh week, mice were challenged intranasally with either DPBS or OVA and injected intraperitoneally with vehicle (DPBS containing 0.01% DMSO), 1 mg/kg fluticasone propionate (FP; cat no. 20703; Cayman Chemical), or 3 mg/kg fluticasone propionate for three consecutive days (Figure 1A). For chronic exposure, newborn male and female mice were intranasally (i.n.) sensitized and challenged with PBS or mixed allergens (MA), which consists of 10 µg *Alternaria Alternata* (Stallergenes Greer, Lenoir, NC)*,* 10 µg *Aspergillus Fumigatus* (Stallergenes Greer), 10 µg *Dermatophagoides Pteronyssinus* (Stallergenes Greer), and 10 µg OVA 3 times per week for 7 weeks. During the last 2 weeks, mice were intraperitoneally (IP) injected with vehicle (PBS containing 0.015% DMSO), 1, or 3 mg/kg FP (FP; cat no. 20703; Cayman Chemical, Ann Arbor, Michigan) on the same day as MA challenge (Figure 1). Lung function and tissue necropsy were performed 24 h after the last allergen challenge. Mice were anesthetized with 90–120 mg/kg ketamine and 5 mg/kg xylazine. Adequate anesthesia was confirmed by pedal reflex.

***Bronchoalveolar lavage harvesting:*** Bronchoalveolar lavage fluid (BAL) was harvested and total and differential cell analyses were performed as previously described (Livraghi-Butrico et al., 2012; Lewis et al., 2021b). Briefly, mouse trachea were exposed and cannulated with a 19 gauge blunt tip cannula. Whole lungs were flushed with PBS. BAL was centrifuged at 300 × g for 5 min. Supernatant was collected and stored at −80°C for cytokine analyses. Cell pellets were resuspended in PBS and used for total and differential cell counts.

***Lung function.*** Lung function was measured using the Flexivent (SCIREQ, Montreal, Quebec, Canada) as previously described (Lewis et al., 2021b). Briefly, mice trachea was exposed and cannulated with a 19-guage blunt tip cannula. Total resistance was measured *via* forced oscillation maneuvers following nebulization of PBS, 6.25, 12.5, 25, and 50 mg/ml methacholine (Millipore-Sigma, St. Louis, MO).

***Cytokine analyses.*** Lung tissue was homogenized in RIPA buffer. U-plex ELISA plates Mesoscale (MSD, Rockville, Maryland) were coated with antibodies against interleukin (IL)-4. Analytes were measured in whole lung homogenates following manufacturer instructions. Cytokine concentrations were calculated using a standard curve. IL-13 levels were measured in cell-free BAL supernatant using Quantikine mouse IL-13 and CCL17 ELISA kits (cat no. M1300CB and MCC170, R&D Systems, Minneapolis, MN) according to manufacturer’s instructions.

***Histopathological analyses:*** Left lung lobes were processed, paraffin embedded, cut into 6 μm sections, and analyzed for airway inflammation and epithelial mucous cell abundance as previously described (Lewis et al., 2021b). H&E-stained slides were blindly scored for degree of immune cell aggregation in the peribronchiolar and perivascular spaces. Lung sections stained with Alcian Blue-Periodic Acid Schiff (AB-PAS) were used to assess epithelial mucous cell abundance. Analyses were performed using ImageJ (NIH) by a blinded investigator.

***α-Smooth Muscle actin Immunohistochemical analyses:*** Left lung lobes were formalin fixed, embedded in paraffin, and sectioned at 6 µm. Immunohistochemical staining and analyses were performed as previously described (Lewis et al., 2021b). Briefly, left lung lobe sections were stained for α-smooth muscle actin. Smooth muscle actin area was analyzed and quantitated using ImageJ (NIH).

***Total antioxidant capacity analyses:*** Whole lung lobes were weighed and homogenized in DPBS. Total antioxidant capacity was measured with Oxiselect Trolox Equivalent Antioxidant Capacity (TEAC) Assay Kit (Cell Biolabs, San Diego, CA) according to manufacturer’s instructions. Results were normalized to lung tissue weight.

***Gene expression analyses:*** Total RNA was extracted from whole lung homogenates using RNAeasy Mini Kit (Qiagen, Germantown, MD) according to manufacturer’s instructions. RNA concentration was measured by spectrophotometer (NanoDrop, Wilmington, DE). cDNA was generated using the Maxima First Strand cDNA synthesis kit (cat no. K1642; ThermoFisher Scientific, Waltham, MA). Primers for mouse genes *Gpx1* (QT01195936), *Cat* (QT0158106), *Sod1* (QT00165039), *Sod2* (QT00161707), and *Tnfaip3* (QT0013464) were purchased from Qiagen. Additional primers for sequences are listed in Table 1. Fold change was calculated using the ΔΔCT method.

**TABLE 1:** Primer sequences.

Primer	Forward sequence (5’ to 3’)	Reverse sequence (5’ to 3’)
Map kinase phosphatase 1 (*Mkp1*)	CTA​CCA​GTA​CAA​GAG​CAT​CCC	CAC​TCT​GCT​GAA​AGG​AGT​CTG​CAC​AA
FK506 binding protein (*Fkbp5*)	GAA​GGG​AAC​TGT​GTA​CTT​CAA​GGG	TCC​AGC​CAG​GAC​ACT​ATC​TTC​CT
Glucocorticoid-induced leucine zipper (*Gilz*)	AGC​CGG​TTT​ACC​TGA​AGT​GG	CAT​CTT​CTC​CGG​CTT​GGA​GG
Glucocorticoid receptor interacting protein 1 (*Grip1*)	CTG​CAT​AGG​TGC​GCG​AAG​AT	ATT​TTA​AAA​CCG​GGC​CTC​GC
S16	TGC​AGG​TCT​TCG​GAC​GCA​AGA​AAA	CGA​ATA​TCC​ACA​CCA​GCA​AAT​CGC

***Statistical analyses.*** Data were analyzed by performing one-way ANOVA with Bonferroni post hoc analysis for multiple comparisons. Data were analyzed and graphed using GraphPad Prism eight Software (GraphPad, La Jolla, CA). Values are presented a mean ± standard error and significantly differences are indicated by *p* < 0.05.

## Results

**Total immune cell and eosinophil infiltration are reduced with corticosteroid treatment.** OVA- and MA-challenged mice exhibited a significant increase in immune cell infiltration in bronchoalveolar lavage (BAL) compared to respective controls (Figures 1B,C). The significant increase in total cells was attributed to an increase in eosinophils in both OVA- and MA-challenged mice (Figures 1D,E). Treatment with 1 mg/kg fluticasone propionate (FP) significantly reduced total immune cell numbers in OVA-challenged mice (Figure 1B), but not MA-challenged mice (Figure 1C). Treatment with 3 mg/kg FP significantly reduced the total number of immune cells in both OVA- and MA-challenged mice (Figures 1B,C). The total number of BAL eosinophils was significantly reduced with 1 and 3 mg/kg FP treatment in both OVA- and MA-challenged mice (Figures 1D,E).

**FIGURE 2:**
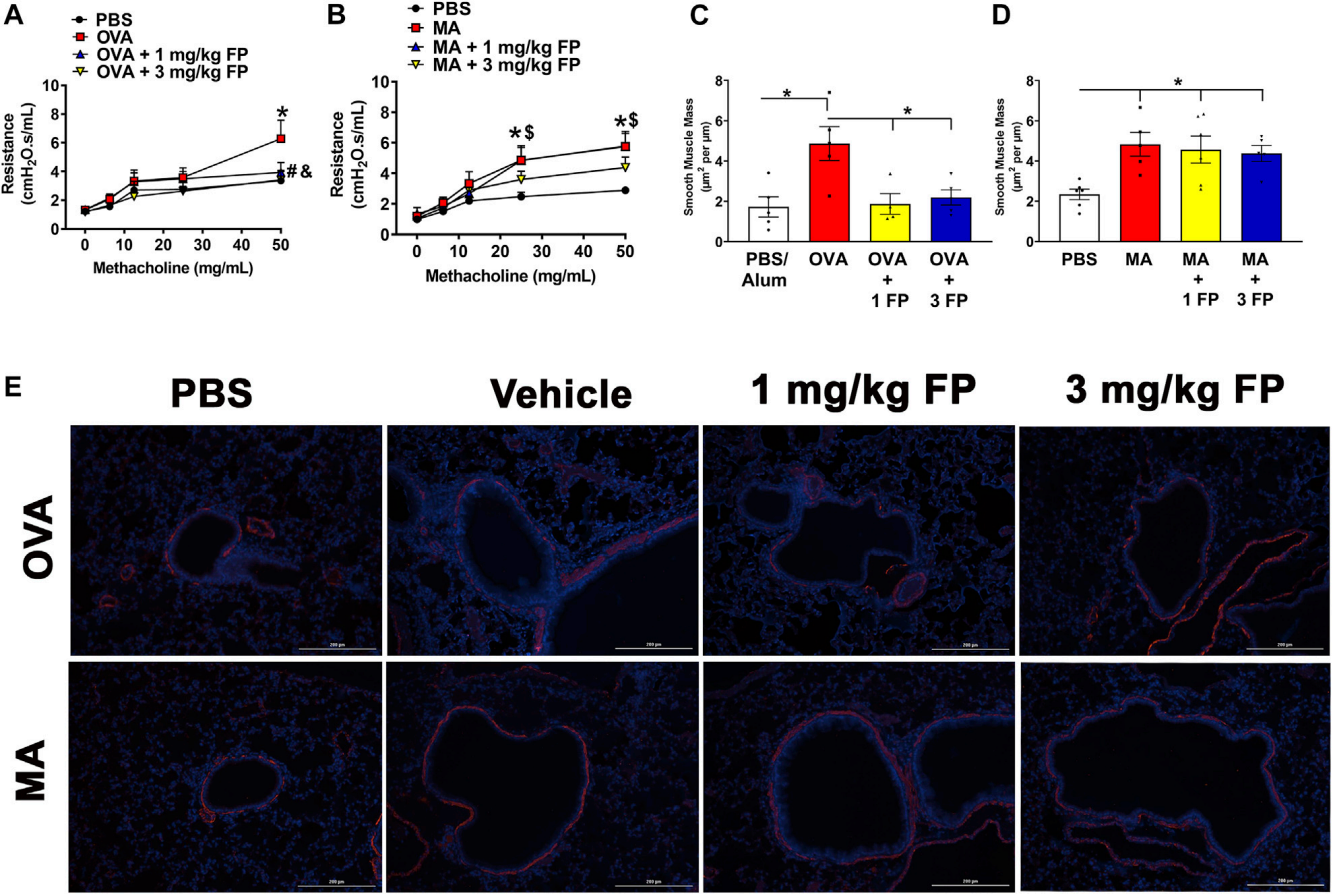
Airway hyperresponsiveness (AHR) and smooth muscle mass are corticosteroid insensitive in MA mice. AHR is significantly increased in **(A)** OVA- and **(B)** MA-challenged mice. Treatment with 1 mg/kg and 3 mg/kg fluticasone propionate (FP) significantly reduces AHR in OVA-challenged mice, but does not affect MA-challenged mice. **(C)** OVA- and **(D)** MA-challenged mice exhibited increased airway smooth muscle mass. Treatment with corticosteroids reduced ASM mass in OVA-challenged, but not MA-challenged mice. **(E)** Representative photomicrographs of left lung lobe sections stained with α-smooth muscle actin. Data are presented as ± SEM, *n* = 4–13 mice per group. *p* < 0.05. For AHR *, # indicates significant difference from PBS. $, and indicates significant difference from OVA-challenged mice. For ASM mass, * indicates significant difference between groups.

**Corticosteroids do not reduce airway hyperresponsiveness (AHR) or airway smooth muscle (ASM) mass in MA-challenged mice.** To assess corticosteroid sensitivity in our mouse models of OVA- and MA-induced airway inflammation, AHR was measured using forced oscillation maneuvers (Flexivent) in response to increasing doses of methacholine (0–50 mg/ml). OVA- and MA-challenged mice exhibited significantly higher AHR in comparison to respective PBS controls (Figures 2A,B). Treatment with 1 and 3 mg/kg fluticasone propionate (FP) significantly reduced AHR in OVA-challenged mice (Figure 2A), but not MA-challenged mice (Figure 2B).

To determine airway smooth muscle (ASM) mass, left lung lobe sections were stained for α-smooth muscle actin expression. OVA- and MA-challenged mice exhibited significantly increased ASM mass compared to PBS controls (Figures 2C,D). Treatment with 1 and 3 mg/kg fluticasone propionate significantly reduced ASM mass in OVA-challenged mice (Figure 2C), however ASM mass was not reduced with either corticosteroid dose in MA-challenged mice (Figure 2D).

**FIGURE 3:**
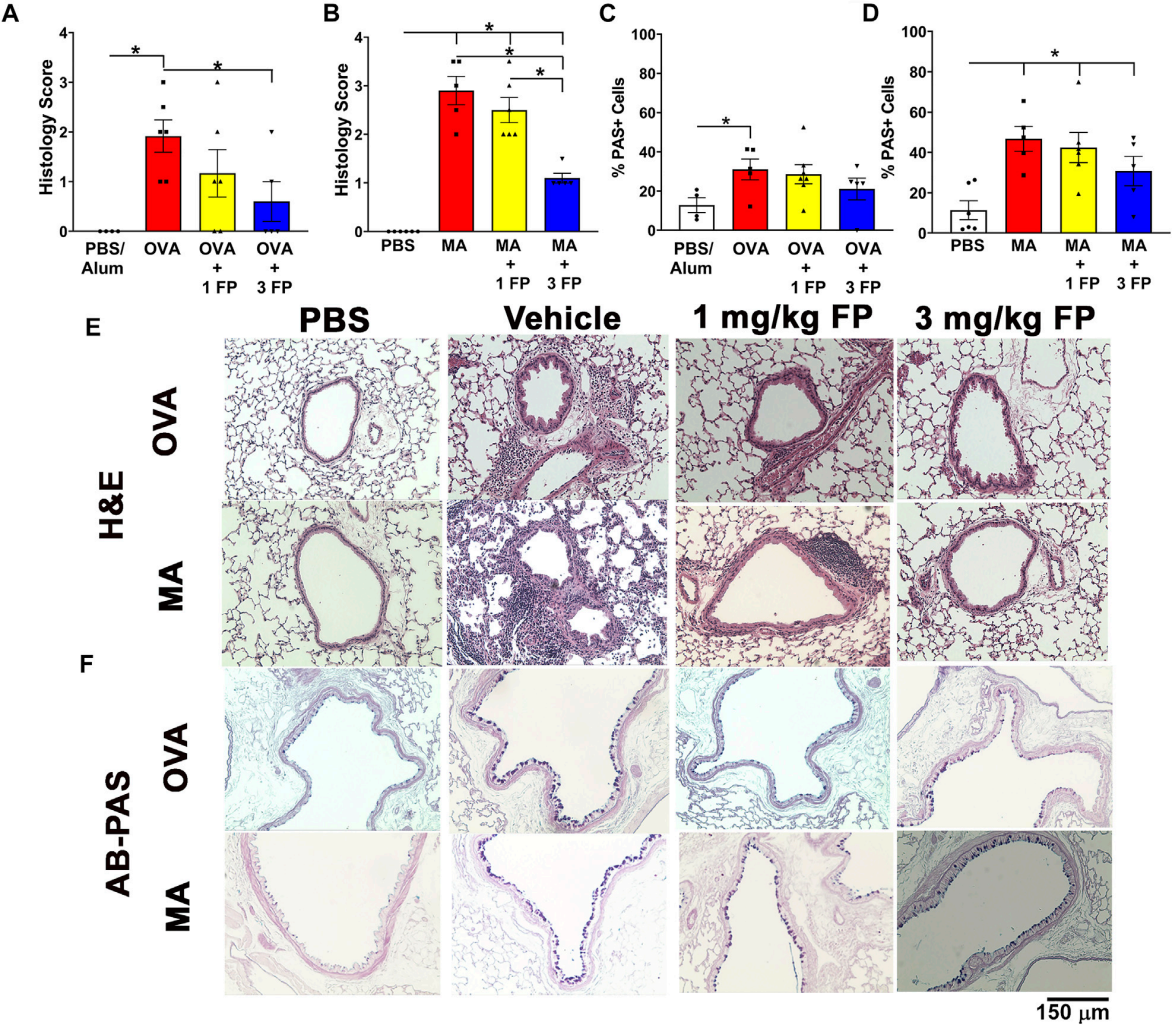
Corticosteroids reduce airway inflammation but not mucous cell abundance in acute and chronic allergic airway inflammation. **(A)** OVA- and **(B)** MA-challenged mice exhibited increased airway inflammation that was reduced with 3 mg/kg FP. **(C)** OVA- and **(D)** MA-challenged mice exhibited increased airway epithelial abundance that is not reduced by corticosteroids. Representative photomicrographs of **(E)** H and E- and **(F)** AB-PAS-stained left lung lobe sections. Data are presented as ± SEM, *n* = 4-7 mice per group. *p* < 0.05. * indicates significant difference between groups.

**Corticosteroids reduce airway inflammation but not epithelial mucous cell abundance in acute and chronic allergen challenge.** To assess the degree of airway inflammation, H and E-stained left lobe sections were scored for peribronchiolar and perivascular immune cell aggregation. OVA- and MA-challenged mice exhibited significantly increased airway inflammation compared to PBS controls (Figures 3A,B). Treatment with 1 mg/kg fluticasone propionate (FP) did not significantly reduce airway inflammation in either OVA- or MA-challenged mice (Figures 3A,B). Airway inflammation was significantly reduced in both OVA- and MA challenged mice when treated with 3 mg/kg FP (Figures 3A,B).

Increases in epithelial mucous cell abundance are key in allergic airway inflammation (Curran and Cohn, 2010). To analyze for airway epithelial mucous cell composition, lung sections were stained with AB-PAS and PAS + cells were quantified. Compared to PBS controls, OVA- and MA-challenged mice exhibited a significantly higher percentage of mucous cells in the airway epithelium (Figures 3C,D). Treatment with 1 mg/kg and 3 mg/kg fluticasone propionate did not significantly reduce the abundance of mucous cells in OVA- and MA-challenged mice (Figures 3C,D).

**FIGURE 4:**
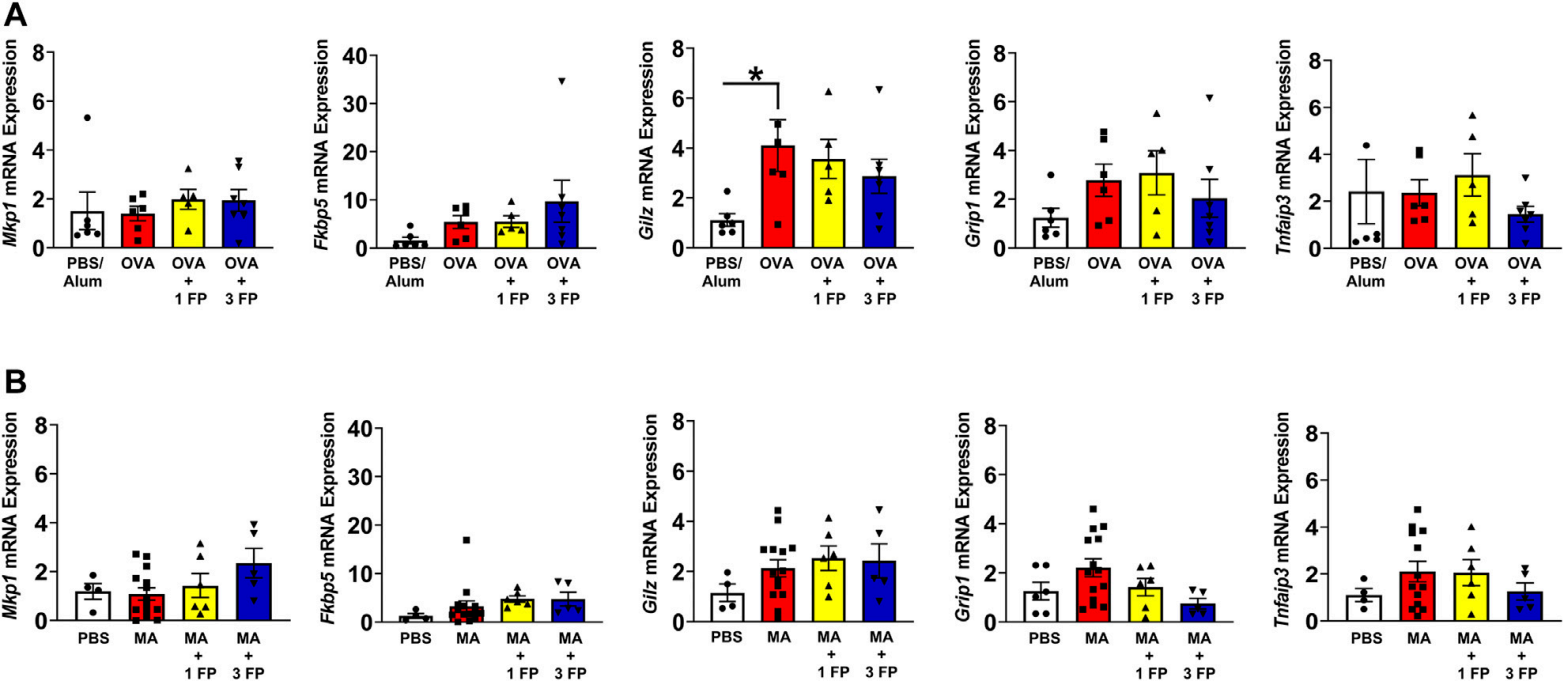
Glucocorticoid receptor (GR) target genes are not altered in OVA- and MA-challenged mice. **(A)** OVA-challenged mice exhibited higher mRNA expression except *Gilz* expression. **(B)** There were no significant differences in GR target genes in MA-challenged mice. Data are presented as ± SEM, *n* = 4–14 mice per group. *p* < 0.05. * indicates significant difference between groups.

**Glucocorticoid (GR) target gene and antioxidant gene expression in OVA- and MA-challenged mice.** To assess GR gene expression, qPCR was performed on whole lung homogenates for expression of GR target genes *Mkp1*, *Fkbp5*, *Gilz, Grip1*, and *Tnfaip3*. OVA-challenged mice exhibited a significant increase in *Gilz* compared to PBS controls (Figure 4A). There were no significant differences in the other analyzed genes regardless of allergen exposure or corticosteroid treatment (Figure 4).

**FIGURE 5:**
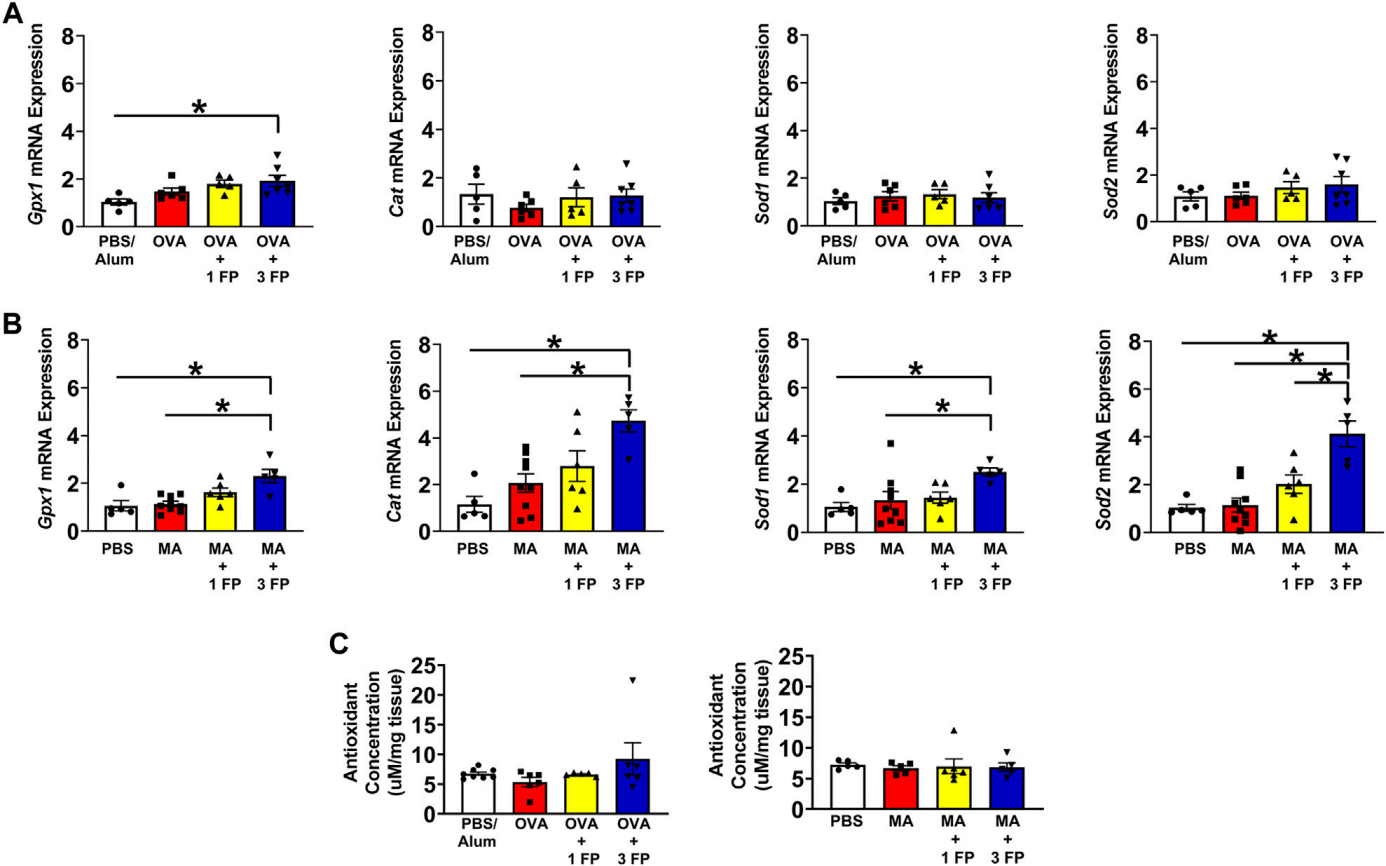
Antioxidant gene expression and total antioxidant capacity. **(A)** OVA- and **(B)** MA-challenged mice exhibited no significant differences in antioxidant gene expression compared to PBS controls. In MA-challenged mice, treatment with 3 mg/kg FP resulted in increased *Gxp1, Cat, Sod1, and Sod2* mRNA expression. **(C)** Total antioxidant capacity remained unchanged in OVA- and MA-challenged mice. Data are presented as ± SEM, *n* = 5–8 mice per group. *p* < 0.05. * indicates significant difference between groups.

Decreases in antioxidant capacity contribute to the development of oxidative stress and potentially corticosteroid sensitivity (Jesenak et al., 2017). To assess differences in gene expression associated with antioxidant responses in the lung, we analyzed whole lung homogenates for *Gpx1, Cat*, *Sod1,* and *Sod2* mRNA expression. Compared to PBS controls, OVA- and MA-challenged mice exhibited no significant differences in antioxidant-related gene expression (Figures 5A,B). However, treatment with 3 mg/kg fluticasone propionate (FP) resulted in higher mRNA expression of *Gxp1* in OVA-challenged mice (Figure 5A). In MA-challenged mice, treatment with 3 mg/kg FP resulted in increased expression of *Gxp1*, *Sod1*, *Cat*, and *Sod2* (Figures 5E–H). We also analyzed whole lung homogenates to assess for total antioxidant capacity. There were no significant differences in total antioxidant capacity regardless of allergen challenge or corticosteroid treatment (Figure 5C).

**FIGURE 6:**
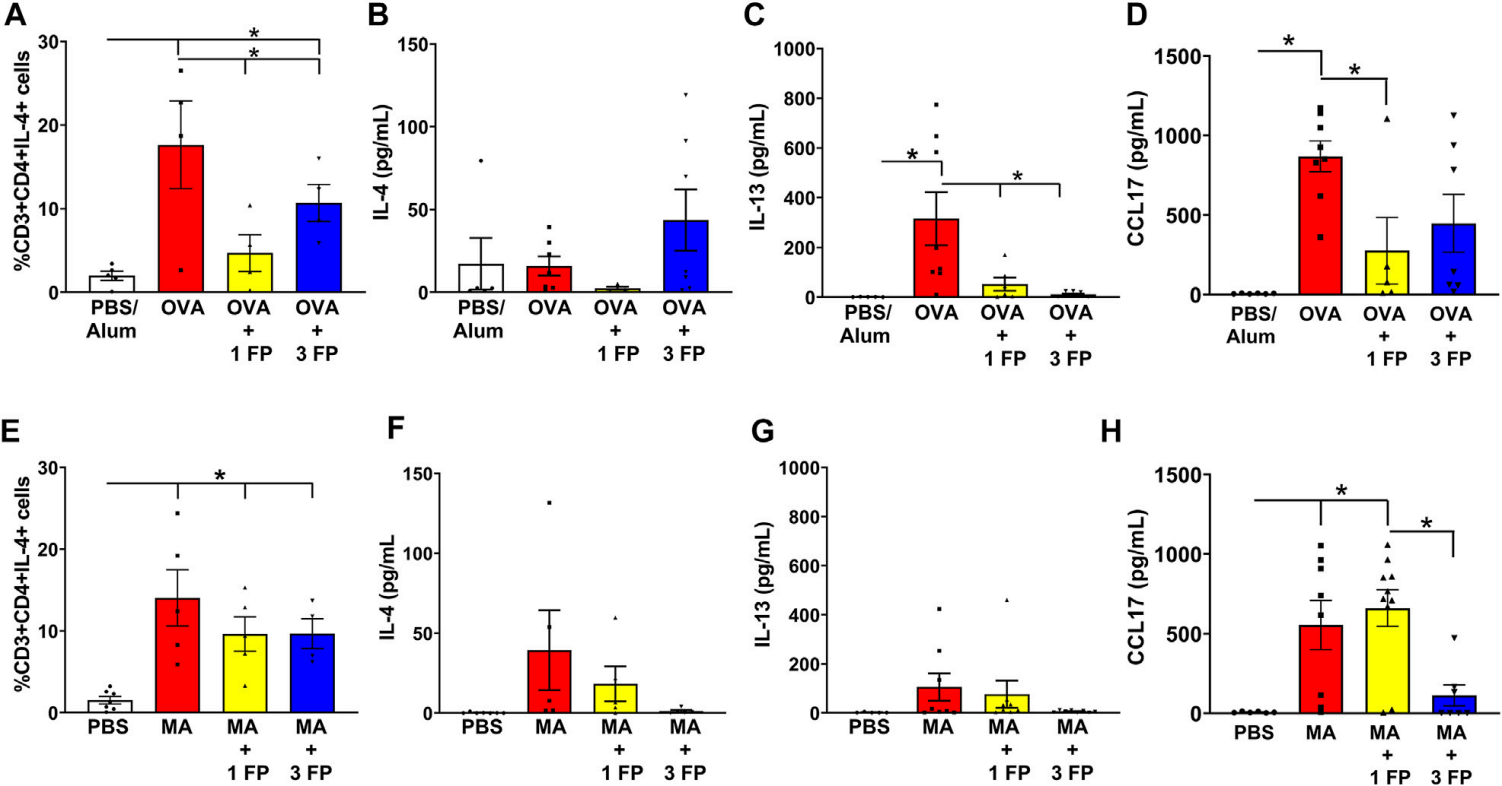
IL-4^+^ Th2 cell populations persist in MA-challenged mice. **(A)** OVA- and **(E)** MA-challenged mice exhibited significant increases in Th2 cell populations. **(A)** Treatment with 1 and 3 mg/kg reduced Th2 cell populations in OVA-challenged mice. **(B)** BAL levels of IL-4 were not significantly increased in OVA-challenged mice. **(C)** BAL levels of IL-13 were significantly increased in OVA-challenged mice and reduced with corticosteroid treatment. **(D)** Fluticasone reduces CCL17 levels in OVA mice. **(E)** Treatment of 1 or 3 mg/kg FP did not reduce Th2 cell populations in MA-challenged mice. **(F)** IL-4 or **(G)** IL-13 levels trended to be increased in MA-challenged mice. **(H)** CCL17 levels remain increased MA treated mice administered fluticasone. Data are presented as ± SEM, *n* = 4–10 mice per group. *p* < 0.05. * indicates significant difference between groups.

**Corticosteroids do not reduce Th2 populations in chronic airway inflammation.** To assess for T-helper 2 (Th) infiltration, a type 2 inflammatory marker, single-cell suspensions generated from whole lungs and analyzed via flow cytometry. Th2 lymphocytes were gated as CD45^+^CD3^+^CD4^+^IL-4^+^ cell populations. Compared to PBS controls, OVA- and MA-challenged mice exhibited a significantly higher percentage of IL-4^+^ Th2 cells (Figures 6A,E). Treatment with 1 mg/kg and 3 mg/kg fluticasone propionate significantly reduced Th2 cell populations in OVA-challenged mice (Figure 6A), but not the MA-challenged mice (Figure 6E). IL-4 levels were trending to increased expression levels in OVA- or MA-challenged mice (Figures 6B,F). OVA-challenged mice exhibited significant increases in BAL IL-13 levels that was significantly decreased with 1 and 3 mg/kg FP (Figure 6C). Although trending higher, there were no significant differences in IL-13 levels in MA-challenged mice (Figure 6G). Expression of CCL17, a type 2 chemokine, was significantly increased in OVA and MA challenged mice. CCL17 expression was reduced by 1 mg/kg FP in OVA mice but not MA mice (Figures 6D,H).

## Discussion

Although type 2 inflammation is commonly associated with corticosteroid-sensitive mild asthma, individuals with severe asthma and corticosteroid insensitivity can also exhibit a predominantly type 2-high inflammatory endotype that is insensitive to corticosteroids (Ray et al., 2020). To understand corticosteroid sensitivity in models of type 2 allergic airway inflammation, we administered a corticosteroid dose response in acute (OVA) and chronic (MA) allergen models. Our data indicate differential responses between the OVA and MA models, with the OVA model inducing airway inflammation, AHR, and airway remodeling that is more corticosteroid sensitive.

In the present study, we compared corticosteroid sensitivity of allergen mouse models used to induce allergic airway inflammation. OVA sensitization and challenge are widely used to induce allergic airway inflammation (Daubeuf and Frossard, 2013), however OVA is not associated with human asthma. In more recent years, studies have adopted using inhaled allergens that contribute to human asthma, including ragweed, cockroach, house dust mites (HDM), *Aspergillus fumigatus*, and *Alternaria alternata* (Nials and Uddin, 2008). OVA and common allergens induce similar allergic airway immune responses in mice by increasing robust type 2-associated inflammation with Th2 cell populations, type 2 cytokines, and eosinophil infiltration (Choi et al., 2016; Kim et al., 2019). Despite these similarities in type 2 inflammation, persistent immune cell infiltration, AHR, remodeling, and mucous cell metaplasia are more prominent following repeated or chronic exposure with common allergens (Zosky et al., 2008; Zoltowska et al., 2016; Dullaers et al., 2017; Chen and Wang, 2019). Previous studies show eosinophil lung infiltration and airway remodeling in mice remains increased for up to 7 weeks post-exposure to HDM, but not OVA (Johnson et al., 2004). Inhalation of common allergens contain proteases, endotoxins, and chitin that enable them to induce immunological and physiological responses in the lung. These exposures induce airway epithelial injury, with IL-25, IL-33, and thymic stromal lymphopoietin (TSLP) production to initiate type 2 airway inflammation further enhancing the Th2-associated immune response (Leino et al., 2013).

In contrast to the acute model, MA-challenged mice exhibited persistent airway inflammation and remodeling in terms of immune cell aggregation in the peribronchiolar/perivascular spaces and airway smooth muscle (ASM) mass. Immune cell composition in OVA and MA models were similar, with eosinophil and lymphocyte airway infiltration. However, we observed that eosinophil levels in BAL were blunted by low dose corticosteroids in OVA mice, while MA mice need a greater corticosteroid dose to significantly reduce eosinophils. We also found that pathological and structural changes persisted in MA-challenged mice treated with corticosteroids, while they resolved in treated OVA-challenged mice. Consistent with previous studies (Lee et al., 2008), we found that low dose corticosteroids were highly effective in reducing AHR and ASM mass in OVA-challenged mice. Type 2 inflammation also affects airway structure and function by promoting mucous cell metaplasia and mucus hyperproduction (Rogers, 2004). We observed that both OVA- and MA-challenged mice exhibited persistent epithelial mucous cell abundance despite treatment with corticosteroids. These findings are in contrast with prior studies showing that corticosteroids reduce mucous cell hyperplasia in OVA and HDM models (Southam et al., 2008; Yao et al., 2010). Differences in allergen challenge and corticosteroid treatment protocols could account for the difference in our study and previous reports. Collectively, our data indicate differences in corticosteroid sensitivity of airway inflammation and remodeling between our OVA and MA models.

Corticosteroid insensitivity has been attributed to alterations in glucocorticoid receptor (GR) expression and diminished GR activity, enabling airway inflammation to persist in severe asthma (Barnes, 2013; Lewis et al., 2021a). To assess GR activity and its relationship to corticosteroid sensitivity, we measured expression of anti-inflammatory and antioxidant genes known to be regulated GR. Among the anti-inflammatory genes, including established GR target genes, such as *Dusp1*, *Fkbp5*, and *Gilz*, we did not observe significant changes. However, *Gilz* was found to be significantly increased in OVA challenged mice. It is important to note that these anti-inflammatory GR target genes can also be enhanced by pro-inflammatory signaling pathways (Van Bogaert et al., 2010; Gerber et al., 2021), potentially contributing to the lack of significant changes in lung tissue. Conversely, we found multiple antioxidant genes to be increased by allergen-challenged and corticosteroid treated mice. Glutathione peroxidase 1 (*Gpx1*) was increased by FP in both OVA and MA models. We also observed significant increases in catalase (*Cat*) and superoxide dismutase 1 and 2 (*Sod1* and *Sod2*) mRNA expression in MA mice. GR has been shown to regulate glutathione synthesis (Obrador et al., 2014) and *Gpx3* expression in lung cancer cells (An et al., 2016), highlighting its ability to contribute to antioxidant responses. Despite increased antioxidant gene expression, we did not observe changes in overall lung tissue antioxidant capacity. These data suggests that GR is functional in both OVA and MA models, an interpretation supported by reduced eosinophil BAL counts. We postulate that corticosteroid insensitivity in the chronic MA model is not due to disruption in GR signaling or antioxidant responses.

In addition to airway inflammation and airway smooth muscle mass, IL-4^+^ Th2 cell populations were found to be insensitive to corticosteroids in the chronic MA model. This is consistent with our recent study showing corticosteroid sensitivity in IFNγ^+^ Th1 cells, but not IL-4^+^ Th2 or IL-17A^+^ Th17 cells (Lewis et al., 2021b). These findings suggest that Th cell subsets may have differential corticosteroid sensitivity. Corticosteroids modulate Th cell populations and effector functions by GR-induced apoptosis and suppression of effector cytokine production (Banuelos and Lu, 2016). Mouse Th2 cells have been shown to be resistant to corticosteroid-induced apoptosis through enhanced BCL-2 expression, although their IL-4 production was corticosteroid-sensitive (Banuelos et al., 2016). The reason for the discrepancy in number of IL-4^+^ Th2 cells in our OVA and MA models is not fully clear. CCL17 expression is increased by type 2 cytokines, IL-4 and IL-13, and is an important mediator in type 2 responses (Faffe et al., 2003; Bisset and Schmid-Grendelmeier, 2005). Production of CCL17 could be attributed to increased release by airway epithelial cells that lack a negative regulatory mechanism (Zissler et al., 2016). In contrast to OVA mice, we found that CCL17 expression remained increased in MA mice treated with 1 mg/kg fluticasone. CCL17 promotes Th2 cell recruitment by binding CCR4 and contributes to type 2 inflammation by increasing eosinophils, AHR, and IL-4 expression (Zhang et al., 2017). In human asthma, CCR4+ Th2 cells are increased and correlate with asthma severity (Kurashima et al., 2001; Kurashima et al., 2006). The inability for corticosteroids to reduce CCL17 levels could contribute to persistent Th2 cell recruitment, suggesting MA mice may have CCR4+ Th2 cells. The question remains if CCR4 inhibition would enhance corticosteroid sensitivity in MA mice. The persistence of Th2 cells in MA mice could also involve lack of negative regulator signals that counter Th2 pathways, e.g. IFNγ, or Th2 cell resistance to apoptosis (Huang et al., 2001; Williams et al., 2018). Additionally, ILC2 activation, which promotes type 2 inflammation, could also contribute to enhanced allergic airway inflammation and corticosteroid insensitivity (Luo et al., 2019). ILC2 regulation involves negative regulatory signals from IFNγ or PGE_2_ (Duerr et al., 2016; Maric et al., 2018; Zhou et al., 2018; Zhou et al., 2020). While PGE_2_ inhibits ILC2 expansion through its receptors EP2 and EP4 (Maric et al., 2018; Zhou et al., 2018), PGE_2_ may also enhance ILC2 pro-inflammatory effector functions through EP3 (Jakwerth et al., 2021). Additional studies are needed to better understand why Th2 cells, and potentially other type 2 immune cells, persist in the MA but not OVA model.

Previous acute and chronic mouse models have enabled extensive characterization of airway inflammation and remodeling, but the allergen sensitization and challenge likely has an impact on corticosteroid sensitivity (Lloyd et al., 2001; Ellis et al., 2003; Kumar et al., 2008; Nials and Uddin, 2008). Mouse models of acute allergic airway inflammation with exposure to OVA or HDM exhibit increased type 2 cytokine production, eosinophilic infiltration, and airway hyperresponsiveness (Tournoy et al., 2000; Lloyd et al., 2001; Nials and Uddin, 2008; Collison et al., 2011). These acute models demonstrate similar corticosteroid sensitivity, with suppression of airway inflammation and AHR (Collison et al., 2011; Hu et al., 2018). Conversely, chronic challenge with HDM induces type 2 inflammation with more persistent airway remodeling (Ellis et al., 2003; Ulrich et al., 2008; Woo et al., 2018). More recently, prolonged administration of multiple allergens has been shown to induce corticosteroid insensitivity in mice (Duechs et al., 2014) and may be more reflective of the complex allergen sensitization experienced in allergic asthma (Roberts et al., 2020). In addition to OVA and HDM, we administered *Aspergillus fumigatus* and *Alternaria alternata* extracts, which are fungal allergens associated with corticosteroid insensitivity in severe asthma (Castanhinha et al., 2015; Fraczek et al., 2018; Bush, 2020). These fungal allergens enhance airway inflammation through activation of innate and adaptive type 2 responses (Iijima et al., 2014; Bartemes and Kita, 2018), implicating *Aspergillus fumigatus* and *Alternaria alternata* in the persistent IL-4^+^ Th2 cells we observed in MA-challenged mice.

Comparing OVA and MA models by performing a dose response with corticosteroids in both models, enabled us to assess corticosteroid sensitivity. Our approach is in contrast to studies investigating corticosteroid insensitivity or resistance, which typically challenge with one allergen and treats with corticosteroids at one dose. A key limitation is the individual contributions of HDM, *Aspergillus fumigates,* and *Alternaria alternata* to corticosteroid insensitivity were not determined. Studies show that sensitization to more than one allergen, particularly fungal allergens, are associated with corticosteroid insensitivity in patients with difficult-to-treat or severe asthma (Bush, 2020; Roberts et al., 2020; Mistry et al., 2021). Airway inflammation and AHR persist in multiple allergen models despite corticosteroid treatment (Duechs et al., 2014; Zhou et al., 2017), suggesting that these may be more reflective of severe asthma. Nonetheless, additional studies will be needed to further interrogate the effects of different allergens on corticosteroid sensitivity. We also acknowledge important limitations including the lack of characterization of innate and adaptive type 2 immune cell populations. IL-13^+^ Th2 and ILC2 cells will need also need to be examined. Corticosteroid insensitivity in type 2-high asthma is heterogenous and may involve other factors, such as environmental exposures or infection. Incorporating additional factors into allergen models will be important to understanding how corticosteroid sensitivity is affected in type 2 inflammation.

Understanding mechanisms related to type 2-high severe asthma is currently limited by lack of characterization of corticosteroid sensitivity in allergen models. In mice, corticosteroid insensitivity is commonly achieved by augmenting allergic airway inflammation with a pro-inflammatory stimulus/insult or pathogen (e.g., lipopolysaccharide, cyclic-di-GMP, *Hemophillus influenzae*, ozone). Although important, these models induce non-type 2 immune pathways such as Th1 and/or Th17 inflammation, creating asthma endotypes that are distinct from type 2-high severe asthma. We found that chronic MA challenge induced corticosteroid insensitivity that was accompanied by persistent airway smooth muscle mass and IL-4+ Th2 cell populations. These findings have implications for understanding asthma endotypes that are type 2-high and insensitive or refractory to corticosteroids. The mixed allergen model may provide an approach to understand the complex type 2 immune response in severe asthma and opportunities to identify biomarkers that inform on corticosteroid insensitivity. Further investigations into the cellular and molecular differences between acute and chronic models could advance our understanding of mechanisms that contribute to corticosteroid insensitivity in type 2-high severe asthma.

## Data Availability

The original contributions presented in the study are included in the article/Supplementary Materials, further inquiries can be directed to the corresponding author.
